# Should treatment of hypogammaglobulinemia with immunoglobulin replacement therapy (IgRT) become standard of care in patients with chronic lymphocytic leukemia?

**DOI:** 10.3389/fimmu.2023.1062376

**Published:** 2023-04-14

**Authors:** Alessandro Noto, Ramona Cassin, Veronica Mattiello, Marta Bortolotti, Gianluigi Reda, Wilma Barcellini

**Affiliations:** ^1^ Hematology Unit, Fondazione IRCCS Ca’ Granda Ospedale Maggiore Policlinico, Milan, Italy; ^2^ Department of Oncology and Hematology Oncology, Faculty of Medicine and Surgery, University of Milan, Milan, Italy

**Keywords:** hypogammaglobulinemia, CLL, immunoglobulins, IGRT, infections, SARS-CoV-2

## Abstract

Hypogammaglobulinemia (HGG) is a frequent finding in patients with hematological malignancies, and is commonly described in chronic lymphocytic leukemia (CLL) before or after treatment. We reviewed published literature available online in the last thirty years through Medline search of indexed articles focusing on the main differences and advantages of the products now available on the market, namely intravenous Ig (IVIg) and subcutaneous Ig (SCIg) preparations. IgRT is effective and safe in the prophylaxis of infections in a selected group of patients with CLL and hypogammaglobulinemia and is therefore a valuable tool for clinicians in the everyday management of infectious risk. We encourage the use of SCIg formulations as they appear to have similar efficacy but better cost-effectiveness and tolerability.

## Introduction

Hypogammaglobulinemia (HGG) is defined as a reduced concentration of immunoglobulin G (IgG) and/or immunoglobulin A (IgA) in the serum (while immunoglobulin M levels may vary) and is a well-known condition present in hematological malignancies, commonly observed in chronic lymphocytic leukemia (CLL) (1).

HGG occurs in around 25% of CLL patients at diagnosis (2, 3) and reaches 85% of cases during disease course (4), depending on Binet/Rai stage. HGG exposes patients to an increased risk of infectious events and it is not reversible over time. Infections represent the primary cause of death, as they account for 25-50% of mortality rate in this group (5, 6).

For these reasons, it has been hypothesized that CLL patients with HGG could benefit from antibody replacement therapy (IgRT) (7). However, it is still a matter of debate if a direct relation exists between HGG and infectious risk, as some studies have postulated they may be merely concomitant events (8).

With regard to low Ig levels, CLL patients always show peculiar defects of the adaptative immune system (affecting amount and activity of both B-cells and T-cells) (5, 9, 10) which result in increased infection susceptibility. Studies evaluating IgRT were conducted largely before the chemo-immunotherapy and targeted agents’ era (11); therefore, the effectiveness of replacement therapy needs new validation now that small molecule inhibitors have entered clinical practice.

We conducted a narrative review of twenty-one studies, discussing the evidence and practical approach for IgRT in CLL and other lymphoproliferative disorders (LPD) and focusing on its use for the management and prevention of infectious events.

## Infections in CLL: from past to present

The clinical course of CLL is often burdened with recurrent and severe (grade 3/4) infec events (12, 13). “Recurrent” events are defined as infections occurring ≥ 3 times over a year (14). According to Common Terminology Criteria for Adverse Events (CTCAE) vers 5.0, grade 3 infections are classified as those where IV antibiotic, antifungal, or antiviral therapy is indicated, while in grade 4 events urgent intervention is deemed necessary for life-threatening consequences and requires hospitalization (15).

In **Table 1** we reported infectious incidence in some of the largest clinical studies enrolling CLL patients.

**Table 1:** Incidence of infectious events in cll clinical studies.

Study	Study type	Treatment	Patient n	Total infections (%)	Infections G ≥ 3 (%)
Hensael M et al. (16)	Observational retrospective	Miscellaneous	Total: 187 Untreated: 84	80.2 24.5	19.8 3.3
CLL10 (17)	Randomized Prospective (first line)	FCR BR	Total: 561 FCR: 282 BR: 279	84.2 59.1	39.0 26.0
Resonate-2 (18)	Randomized Prospective (first line)	Ibrutinib Chlorambucil	Total: 267 Ibrutinib: 135 Chlorambucil: 132	17.0 17.0	2.0 2.0
CLL-14 (19)	Randomized Prospective (first line)	G-Ven G-Chl	Total: 561 Ibrutinib: 135 Chlorambucil: 132	NA NA	17.5 15.0
Resonate (20)	Randomized Prospective (R/R)	Ibrutinib Ofatumumab	Total: 391 Ibrutinib: 195 Ofatumumaab: 196	36.0 22.5	12.0 15.0
Murano (21)	Randomized Prospective (R/R)	Ven-R BR	Total: 382 Ven-R: 194 BR: 188	44.2 45.2	21.8 17.5

FCR, Fludarabine; Cyclophosphamide, Rituximab; BR, Bendamustine, Rituximab; G-Ven, Obinutuzumab-venetoclax; G-Chl, Obinutuzumab-chlorambucil; R/R, Relapsed/Refractory; Ven-R, Venetoclax-Rituximab. NA, Not Available.

Considering etiology, lower respiratory tract infections by encapsulated bacteria represent a preferred site (22), although soft tissues and gastrointestinal tract have also been described as a source of infection (15). More specifically, isolation of *Streptococcus* and *Haemophilus spp* in the respiratory tract is a common finding in patients with IgG HGG. The predominant involvement of this district might be associated with low IgA and IgG4 serum levels, together with an impaired secretion of mucosal Igs (22).

Even if less frequent, increased risk of viral disease (such as Herpes Simplex Virus, Epstein–Barr virus and Human Herpesvirus 8) has been reported as well (15).

A certain spectrum of infectious complications has been observed after specific therapeutical agents (23). In patients receiving purine analogues, known to induce quantitative/qualitative T-cell deficit, a large group of uncommon pathogens (including opportunistic fungi, Pneumocystis jeroveci, Listeria monocytogenes and Mycobacteria spp) have been described (24). Previous use of alemtuzumab was especially complicated by cytomegalovirus (CMV) reactivation, occurring in 10-25% of patients (23). The long-term infectious impact of new targeted drugs such as B-cell receptor (BCR) signaling pathway inhibitors, B-cell leukemia/lymphoma-2 (Bcl-2) antagonists, and Chimeric Antigen Receptor (CAR) T-cells is yet to be established. Therefore, as new treatment approaches are being developed for CLL, clinicians will have to keep in mind the potentially increased rate of certain infectious complications during therapy (23).

Ibrutinib and second generation Bruton tyrosin-kinase inhibitors (BTKi) have mainly been associated with respiratory tract events due to bacterial but also invasive fungal etiology. In the RESONATE trial (which compared ibrutinib to ofatumumab in patients with relapsed/refractory CLL) a similar incidence of severe infections (mostly lower respiratory and urinary tract infections) in both groups (15% vs 12% of patients) was shown (20). In comparison, the grade of infectious events was significantly lower in treatment-naive patients, as observed in the RESONATE-2 trial (ibrutinib vs chlorambucil) (18). However, a decline in infection rate could be observed during prolonged ibrutinib therapy (25) and a recent study has reported a reconstitution of humoral immune function (mainly IgA) at 12 months after ibrutinib start, possibly explaining lower infection rates (26). The exact cause of this Ig stabilization/improvement remains as fascinating as it is unknown (27).

Idelalisib carries a higher risk of Pneumocystis jiroveci infection and CMV reactivation, whereas pneumonias and febrile neutropenias had an incidence of respectively 6% and 5% in a study evaluating R/R CLL patients treated with Venetoclax (28).

Although small molecule inhibitors have demonstrated unprecedented efficacy on disease burden with manageable side effects, the inhibition exerted on B-cell signaling and other pathways may justify an increase in infectious risk that, therefore, needs to be expected.

However, the actual severity and long-term effects of the cell-mediated and humoral immune alterations produced by these new drugs is yet to be determined.

No studies are available concerning IgRT selectively in patients treated with novel agents.

Overall, the spectrum of different infectious etiologies is highly variable for these patients and is dependent mainly on disease stage and type of previous therapies.

Considering SARS-CoV-2 pandemic, a higher risk for infection has been hypothesized, as the well-known defect in mucosal immunity may hinder virus clearance from upper airways. On the other side, however, CLL-inherent immunodeficiency may act as protection against severe forms of SARS-CoV-2 pneumonia, limiting the inflammatory cascade that is believed to be crucial for COVID-19 related respiratory failure (29–31).

## Hypogammaglobulinemia

HGG is related to both disease-related immune defects and CLL-directed therapy.

Immunoglobulins (Igs) are secreted by plasma cells after exposure to certain environmental antigens, and are categorized into five classes that differ in heavy chain (32). Each isotype plays a specific role and deficiency in each of them leads to different immune dysregulations.

IgM is the most efficient complement-fixing immunoglobulin and acts mainly in acute responses to microbial pathogens. After that, Ig class switch occurs, leading to IgG predominance, protective in the acute infectious phase in the long-term through generation of memory B cells.

IgGs are the most represented plasma Igs, and are further divided into four subclasses: IgG1, IgG2, IgG3 and IgG4. Deficiency in each subclass is observed in 28%, 19.3%, 52% and 22.7% of CLL patients respectively (33). The IgG subclasses differ in their constant region, which is responsible for binding C1q and Fc-receptors, thus performing different effector functions (34). IgG1s, the most abundant subclass, are considered important for protection against Haemophilus influenzae B (35). IgG2 are involved in bacterial capsular polysaccharide antigens response (36) and their deficit is often concomitant with deficiency in IgG4 and/or IgA (37); in addition IgG2 subclass deficiency is associated with increased susceptibility to invasive respiratory infection caused by Streptococcus pneumoniae and Hemophilus influenzae in children and in the setting of allogeneic hematopoietic stem cell transplant.

In patients with CLL, single IgG subclass deficiency is a common phenomenon and, particularly, low levels of IgG3/4, the most frequently involved, are associated with a 100% chance of infection as compared to 50% of overall HGG (33).

Secretory IgA provide a primary defense mechanism against some mucosal infections, while IgE are primarily produced during parasitic infections (38).

In healthy individuals, normal Ig levels exert multiple fundamental anti-infective functions. In bacterial infections, Igs are able to bind toxins and membrane antigens, thus allowing opsonization that is further responsible for bacteriolysis through complement activation. In viral cases, Igs are able to inhibit viral entry into host cells, stimulate antibody-directed cell-mediated cytotoxicity (ADCC) operated by NK cells and promote virus neutralization, alone or through complement-mediated action (39).

In addition, other studies have suggested a potential immunomodulatory action of antibodies, reducing damage produced by inflammatory responses in later stages of infections (40, 41).

As it might be expected, low Ig levels are associated with impaired B- and T-cell function (42). Clonal CD5+ CLL cells have both a suppressive action on the secretions on Igs (43) and a role in inducing a direct apoptosis of healthy Ig-producing CD95+ plasma cells (44), *via* interaction with CD95L on CLL B-cells (45). Moreover, an impaired interaction between CD40L, expressed on CLL cells, and CD40 expressed on T-lymphocytes has been reported to hinder normal differentiation of non-malignant B cells, resulting in a defect of IgG and IgA class-switch (46, 47). Even at an early stage of disease, a deficiency of helper T cells together with an increase in the number of suppressor T cells can be responsible for reduced Ig production (42).

Treatment of the underlying LPD may also add complexity to this scenario (16). As a matter of fact, treatment with anti-CD20 agents can lead to persistent HGG in up to 6.6% of patients (48), while 38.5% experience transient HGG (2) associated with increased infectious risk (48). Moreover, chemo-immunotherapy regimens with purine analogues and alkylating agents, are all but beneficial on CLL-related HGG, conferring additional immunosuppresion to a pre-existing immunodeficit (49).

As already mentioned, an association between HGG and infections has been reported, especially when IgG levels are lower than 6 g/L (15), although this observation is not confirmed in all studies (50). Indeed, not all CLL patients with HGG will develop severe infections, and normal levels of Igs are not necessarily protective against infectious risk. Moreover, no consistent correlation with a specific immunoglobulin class deficiency has emerged (51, 52).

The basis for such discordance is probably multi-factorial and may be related to the complex disease-induced immune dysfunction.

## Immunoglobulin replacement therapy (IgRT)

### Product description

IgRT consists in the intravenous (IV) or subcutaneous (SC) supplementation of normal polyvalent IgG antibodies derived from plasma of healthy donor (from a minimum of 1000 donors, typically including a larger number (53) ranging from 3000 to 60000 donors) (54).

IgG is the primary and most active constituent of all formulations, but different product vary in their pharmaceutical properties (eg stabilizers; additives; levels of impurities; IgG monomer, dimer, and aggregate concentrations; content of IgA and IgM (55)). IV products reflect normal serum hierarchy including mainly IgG1 and IgG2, with a lower amount of IgG3/4 subclasses. IVIg and SCIg formulations contain approximately 290 mOsm/kg (range 208-1250 mOsm/kg) in most cases, that is within range of physiologic values (55). Deviations from such values may indeed put patients at higher risk for infusion-related adverse events (AE) as well as thrombosis or aseptic meningitis in patients with predisposing factors (advanced age, cardiac/renal failure) (55, 56). The pH of IgRT products should be considered before infusion since the presence of product pH considerably below physiologic levels may trigger a reaction at injection site. Therefore, although pH of approved product usually ranges from 4 to 7.2 (55), a longer time of infusion should be adopted for formulations with pH < 5.

Different concentrations of other isotypes (namely IgA), can be present in IgRT products, lower in IVIg than SCIg preparations (<1-200 mcg/mL versus 37-80 mcg/mL respectively) (55). As cases of severe anaphylaxis have rarely been reported in patients with severe IgA deficiency harboring anti-IgA IgE antibodies the administration of low-IgA formulations seems to show a better safety profile in those who have previously experienced severe allergic reactions (55).

With SCIg infusions the product is gradually released into systemic circulation without a post-infusional peak that is typical of IVIg. Therefore, as reported in some cases, SCIg may be administered safely in IgA-deficient patients who have experienced AEs to IVIg products (55–57).

### Route and dosage of IgRT administration

Products used for passive immunization are available in three formulations: Igs for intramuscular injection (IMIg), IVIg (intravenous) and SCIg (subcutaneous). The first one is generally not indicated for IgRT, but is useful for certain infections (e.g. hepatitis A, measles, and rubella). IVIg products are indicated for the treatment of primary and secondary antibody deficiencies. The target values of serum IgG that should be reached during therapy, however, remains to be established. There are several published experiences in PID underlining the importance of maintaining adequate IgG trough levels when IgRT is used: a meta-analysis including seventeen studies on 676 PID patients treated with IgRT reported that pneumonia incidence declined by 27% with each 100 mg/dl increment in trough IgG and that maintenance of 500 mg/dL IgG trough levels was associated with a 5-fold pneumonia risk than that with 1000 mg/dL (0.113 cases per patient-year vs 0.023) (58). Although different mechanisms are involved in PID and SID (with the former probably requiring higher IgRT dosage to prevent infection occurrence), it reasonable to assume that also in SID IgG trough levels should never be lower than 500 mg/dl.

It is known that dosage should be weight-based, with exception of patients with obesity (59), where adjusted body weight should be preferred to calculate IgRT dose. Some studies (60) did not find a difference between 0.25 and 0.5 g/kg og IVIg every 4 weeks, since no difference in infection frequency between the two groups was observed. Nonetheless, the most recommended dosage for IVIg is 0,4 g/kg every 3-4 weeks (14). Steady state concentration is usually reached after 11–12 weeks (or 4 infusions) (61).

Even if a direct correlation between dosage administered and clinical benefit has not been demonstrated in SID, a higher dosage of IgRT (either IV or SC) might be taken into account to improve outcome: it may be increased if infectious complications persist, whereas a reduction might be possible in those who achieve high IgG trough levels with stable clinical responses or have experienced infusion-related side effects (14). Although not yet widely used (19% to 34% of IgRT in USA and European country) SCIg have received approval for the treatment of all PID, whereas only specific cases of SID (namely CLL, multiple myeloma and HGG post-hematopoietic stem cell transplantation) can be treated with SCIg (HIZENTRA^®^) (62). The infusion usually takes several hours and is delivered by a portable infusion pump using abdomen, arms and thighs as infusion sites. Reduced frequency of systemic side effects allows for self-administration, usually at home with no need for premedication (63).

With IV product use, administered every 3 or 4 weeks, peak concentrations are higher [160% of that obtained by SC infusion (41)] and trough concentrations are lower, which can raise the incidence of systemic AEs and impact tolerability of therapy. Conversely, SCIg infusions are typically administered more frequently (i.e. fortnightly or weekly), resulting in steady state concentrations and consequent higher IgG trough levels compared to same doses of IVIg (64). This allows fewer fluctuations in Ig plasma levels and the added benefit of fewer adverse events.

In recent years recombinant hyaluronidase-facilitated SCIg (fSCIg) have also been introduced in the market. The presence of hyaluronidase permits better absorption and bioavailability of large molecules (such as immunoglobulins) without causing the post-infusional peak of IVIg; moreover, this formulation allows longer infusion intervals (comparable to IVIg) (65).

It is unclear whether the dosage of Ig should be modified when switching from IVIg to SCIg because of differences in pharmacokinetics, therefore most centers tend to administer the same total monthly Ig dosage.

One study reported no change in infection rate in PID patients when switching from IVIg to SCIg (from 817 mg/kg/month IVIg to a lower SCIg dose of 675 mg/kg/month) (66). A dose coefficient of 137% has been added in some countries when switching PID patients from IVIg to SCIg (67). In two other studies, however, patients who changed from IVIg to SCIg reached a significant (p = .01 (14)) rise in serum IgG levels with no change in dosage.

Compagno et al. reported a reduction in infection rate and use of antibiotics in patients affected by LPD while on SCIg, even though they maintained a dosage equivalent to their previous IVIg treatment. SCIg therapy allowed superior benefit when compared to IVIg achieving higher IgG trough levels, lower incidence of infections and reduced need for antibiotics (68). As expected, a lower number of adverse events were registered with subcutaneous infusions, compared to IVIgs, with no serious AEs (68).

Recently, an observational study of 17 patients with SID (including 5 CLL patients) prospectively evaluated IVIg versus SCIg treatment on infection risk, mortality and patient’s perspective (69). The number of infections during the three years of SCIg treatment did not exceed the number of infections during IVIg (69), showing that the two administration routes may have similar clinical efficacy.

Consequently, both modes of administration can successfully elevate serum IgG levels in SID patients and half of the monthly dose should be considered if SCIg administration is preferred as it is delivered twice a month (60).

IVIg administration is generally initially preferred for patient with recurrent/severe pneumonia or for those with concomitant acute conditions such as sepsis (62). SCIg administration may benefit the 10–15% of patients who show increased risk of infection during the 3rd and 4th weeks after receiving IVIg.

On the whole, the choice of IgRT modality should be based on patient characteristics and preferences, as both the IV and SC routes have demonstrated efficacy based on appropriate dosing regimens (57).

### IgRT indication

IgRT has been administrated IV in patients with HGG and/or history of infections since the 1980s (64, 70), reducing frequency of bacterial infections and, as a consequence, related hospitalizations (11).

The expected mechanisms of IgRT efficacy include pathogen opsonization, viral neutralization, toxin inactivation, and complement-mediated bactericidal effects.

Since there is little evidence regarding what serum IgG levels should be reached with treatment, patients with recurrent infections have sometimes been referred for IgRT regardless of their serum IgG levels, using variable dosages (71). Moreover, heterogeneity of patient population in the above-mentioned studies in terms of severity of hypogammaglobulinaemia, frequency of administrations and target IgG levels have added complexity to this scenario.

Data from a few randomized trials in CLL patients with HGG and infection history have inspired the current recommendations for IgRT use and dosage as they demonstrated IVIg effectiveness in significantly decreasing bacterial infection rate and prolonging the time to first infection.

In 1988 the Cooperative Group for the Study of Immunoglobulin in CLL first described the efficacy of IVIg in a randomized double-blind clinical trial (of note, IgG threshold for hypo-IgG was set at 700 mg/dl): in 88 CLL patients IgRT was associated with fewer events of bacterial etiology compared to placebo (24 versus 42; p = 0.01). An early advantage in the experimental arm was confirmed after only 25 days from IgRT initiation. Patients in the IgRT arm did not experience bacterial events for a longer period and this reduction in infection rate was more significant in those who completed one year of therapy (14 vs 36; P = .001) (72). Predictably, IVIg infusions did not have an impact on the frequency of infections of viral (i.e. HSV or VZV) or fungal etiology.

Another crossover study was conducted in the late 1980s to confirm clinical efficacy of IgRT: 12 NHL patients and hypogammaglobulinemia (IgG < 350 mg/dl) or recurrent infections were randomized to receive prophylactic IVIg or placebo every 3 weeks for 1 year and then switch to the other arm (73): a significant lower rate of bacterial infections in the period of IVIg treatment was observed (P = .001).

Few years later, Jurlander et al. (74) confirmed in another study a significant reduction in infection-related hospitalizations and febrile episodes in CLL patients receiving intravenous IgRT.

As for the role of IVIg mitigating the course of sepsis and septic shock in the acute phase no specific guidelines are available for patients with SID (secondary immunodeficit). By comparison, a decrease in mortality has been reported in some series of PID and neonates, this observation needs to be validated in hematological patients (75).

With regard to SCIg administration, fewer studies have evaluated this route of administration in HGG secondary to hematological malignancies. A retrospective Swedish study analyzed antibiotic use and hospitalizations due to infection before and after IgRT with SCIg treatment in 17 patients with SID (14 CLL, 3 NHL patients), showing a decrease in hospital admissions (76). Although limited to a small cohort, this study confirmed that SCIg administration can be as effective as IVIg infusions in hematological SID patients. The second study retrospectively evaluated 61 SID patients (affected by CLL and NHL), who changed administration route (from IVIg to SCIg) confirming efficacy of SCIg in infection rate reduction (68). Thirdly, a retrospective Italian analysis enrolled 131 patients with SID (including NHL, CLL and multiple myeloma patients) to evaluate safety and efficacy of treatment with SCIg for prophylaxis of infections, finding SCIg can be equally effective reducing annual infectious rate in SID patients (14).

More recently, Cinetto et al. (77) retrospectively evaluated efficacy and safety profile of SCIg in SID patients compared to PID patients. From their data, SCIg in SID showed the same efficacy in preventing infections as that in PID, though requiring lower dosage and lower IgG trough levels to achieve infection prevention. Furthermore, they demonstrated SCIg efficacy independently from the underlying cause of SID with a comparable safety profile both in PID and SID.

Lastly, a Greek single-center retrospective study (78) analyzed 33 hematological patients with HGG treated with facilitated-SCIg; the study demonstrated similar efficacy (infection rate 18.1%) and a minor nursery/hospital burden compared to IVIg.

Considering such evidence, the *Guidelines on the core summary of product characteristics (SmPC) for intravenous immunoglobulin (IVIg) products*, recommend regular prophylactic IgRT in CLL patients with severe and/or recurrent infections, inefficacy of antimicrobial treatment and either serum IgG levels < 400 mg/dl or proven specific antibody failure to test immunization (i.e. failure to produce a ≥ 2-fold increase in antibody titre) (14). Frequent measurements of IgG trough levels are encouraged, together with assessment of the incidence of infectious events. CLL patients with mild HGG (400 to 600 mg/dL IgG or with a ≥ 2-fold antibody rise after test immunization), may still benefit from IgRT use if clinically needed (14). Indications on when IgRT should be started, how long it should be continued or when to discontinue it are not available to date.

All things considered, these studies, suggest that SCIg, as already appreciated in PID, represent a valuable option in SID patients regardless of the disease leading to antibody deficiency.

### Adverse events

Reactions to IgRT are divided into local (at the infusion site) or systemic. Systemic reactions may be immediate in 60% of cases (during or within 6 hours of the infusion), delayed in 40% of cases (6 hours - 1 week after infusion) or, rarely, late reactions, occurring > 1 week after the infusion (79). These types of reactions are relatively frequent with IVIg infusions, occurring in as many as 20-50% of patients and 5-15% of all IVIg infusions. Immediate systemic reactions such as head and body aches, chills and fever are usually mild and readily treatable. Immediate anaphylactic and anaphylactoid reactions are uncommon, as are other severe reactions such as TRALI (Transfusion-related acute lung injury).

The most common delayed systemic reaction is persistent headache. Less common, but more serious delayed reactions, include aseptic meningitis, renal failure, thromboembolism, and hemolytic reactions. Late reactions are uncommon but often severe, and include lung disease, enteritis, dermatologic disorders and infectious diseases (79).

Systemic mild/moderate reactions can be managed with premedications such as acetaminophen and antihistamines as needed; steroids are also occasionally used in cases of moderate-to-severe reactions (76).

By contrast, adverse systemic reactions are rare with SCIg administration at home, often by self-infusion, and without premedication (60). On the contrary, local reactions such as persistent pain, bruising, swelling and erythema are rare with IVIg infusions, but common with SCIg infusions. Pain from needle insertion is minimal because non-traumatic needles are used. Nearly 75% of patients have some discomfort associated with swelling and redness at the site of the infusion that usually subside within 24-48 hours (60),. These local effects may lessen with subsequent infusions, making it unnecessary to rotate sites as site-specific tolerance can develop (80). The volume of each infusion can also be reduced by giving smaller doses more frequently, sometimes even daily, without the use of a pump (81). These local adverse effects are rarely severe, and do not usually make patients refrain from continuing with this route.

All in all, the route of administration plays a major role also in the types of AEs seen in patients receiving IgRT, thus influencing route of immunoglobulin prophylaxis administration.

### Sars-Cov-2 era

COVID-19 infection has deeply burdened patients affected by hematological malignancies. Specifically, patients with lymphoproliferative disorders tend to have a more severe course of disease, with reported mortality of up to 37% in the pre-vaccine era (82). A recent multicenter analysis of 366 patients affected by CLL or NHL treated with targeted agents and confirmed COVID-19 between February 2020 and January 2022 has shown how age > 75 years, active hematological disease and severe disease were independent risk factors for COVID-19 mortality, whereas the type of therapy (BTKi vs non-BTKi) had no impact (83). Of note, for CLL patients only age and severity of disease affected OS. Vaccination status deeply affected mortality rate ranging from 39% in unvaccinated patients to 20% in those receiving a booster dose (83) showing the importance of vaccine-induced immunization also in this immunodeficient cohort.

Since the start of SARS-CoV-2 pandemic, some studies have reported the use of IgRT to treat both COVID-19 patients and specifically the autoinflammatory disregulations that is often triggered by uncontrolled SARS-CoV-2 infection. Given the heterogeneity of data collected, it may be difficult to draw definitive conclusions.

One study suggested that high dose IVIg (>15-20 g/day), started precociously, could improve survival and lower the impact of COVID-19 pneumonia, decreasing intensive care and mechanical ventilation needs (84).

Interestingly, some analyses on available IVIg preparations reported the presence of antibodies reacting against SARS-CoV-2 antigens, even though no available data exist on their protective role against COVID-19 infection (30).

SARS-CoV-2 infection in CLL patients appeared to confer a higher risk of severe disease and death (81). Of note, in CLL patients, the presence of low IgG levels did not show a relevant impact on the clinical course of COVID-19 pneumonia, probably reflecting the importance of inflammatory response rather than viral replication in shaping the severity of disease course (29, 85). Nevertheless, IVIg in patients with PID and SID may help to prevent bacterial and fungal superinfections (86), a condition often complicating COVID-19 pneumonia (87, 88).

Moreover, in this setting the role of some targeted agents (i.e. Bruton tyrosine kinase inhibitors) has been proposed as potentially beneficial against severe COVID-19 disease manifestations, possibly due to overall anti-inflammatory, immunomodulatory and anti-platelet activity (31).

As a general practice, switch from IVIg to SCIg self-infusion might be encouraged in order to limit hospital accesses during pandemic peaks.

### IgRT, quality of life and pharmacoeconomics

The increasing use of immunoglobulin prophylaxis over the years has undoubtedly changed health-related quality of life (HRQoL) for a portion of patients with recurrent infections and HGG secondary to malignancy or cancer-directed therapy. Probably due to the chance of less frequent hospital visits and feasible self-administration at home, patient surveys have shown preference for SGIg compared to IVIg infusions (71).

Preliminary data regarding pharmacoeconomic reports on IgRT seem to demonstrate that SCIg retain greater cost-effectiveness than IVIg infusions suggesting that, as both administration routes have similar efficacy in preventing infectious risk, SGIg therapy might be preferred in the treatment of HGG in SID (71).

## Discussion

Infections are responsible for 25-50% mortality rate in CLL and represent the most common complication of CLL-directed therapy. Management of infectious complications can often be challenging for physicians.

In the chemo-immunotherapy era clinicians were well aware of the immunosuppressive role of chemotherapy (especially purine analogues) and monoclonal antibodies (MoAb) (e.g. rituximab, ofatumumab, obinutuzumab, alemtuzumab). In this setting IgRT proved to be an effective tool to maintain adequate levels of serum IgG.

With regard to novel agents (BTKi, BCL2i, PI3Ki) only ibrutinib has shown a potential impact on humoral reconstitution (mainly IgA) that lowered infection rate at 12 months from therapy start in a multicentered study (89), while data on humoral reconstitution during idelalisb or venetoclax are scarce and not encouraging (90).

Even if neutropenia plays a leading role as a risk factor for infections, HGG certainly contributes to expose CLL patients to severe infections and is usually time-progressive and not reversible. It is the most recognized immune defect in CLL and dosage of IgA, IgG and IgM is recommended from International Guidelines during CLL monitoring. However, given the possible correlation between IgG subclass deficiency and infections occurrence, IgG1, IgG2, IgG3 and IgG4 should also be evaluated during patients’ follow-up. As previously shown, single subtype deficit appears to be a better tool than HGG to measure humoral functional dyregulation and select patients at higher risk of for severe infections (33).

Clinical management of infectious risk in patients with SID largely varies among different countries (IgRT alone, IgRT with antibiotic prophylaxis or antibiotic prophylaxis alone). As a general guideline, IgRT is strongly suggested in CLL patients with severe/recurrent infections and HGG, therefore narrowing this indication to a very specific group of patients.

In our practice IgRT is reserved to patients with IgG level < 400 mg/dl and with increased rate of infections (at least 3 events requiring antimicrobial therapy in ¾ 6 months).

The question of the correct dosage of IVIg to prevent bacterial infections is still unresolved and is probably patient-dependent even if a dose of 0.4 g/kg every 3-4 weeks is generally suggested. In our experience, we observed a reduction of infectious events with monthly administrations of 0.4 g/kg of IVIg. Maintenance of trough serum IgG in treated patients above 500–700 mg/dL is a reasonable goal and we sometimes adjust IgIV dosage in order to reach this therapeutic range.

Clinicians need to be aware of preparation differences since they can affect safety and tolerability in some patients. While IVIg appears to be the most frequently used form of IgRT administration (76), SCIg offers a manageable alternative that appears to have similar clinical efficacy but better cost-effectiveness. As a fact, IV administration requires al least 3-4 hours and clinical monitoring due to the risk of infusion-related reactions. Moreover, patients are requested frequent hospital attendance which may certainly impact their HRQoL and subsequent occupation of the outpatient/Day Hospital service. The choices of SCIg and self-infusion has, at our institution, reduced costs and improved HRQoL. Therefore we believe all CLL patients with secondary immunodeficiency requiring IgRT, should have access to SCIg as a treatment option and should be involved in the decision-making of the route of administration (14).

Conversely, IVIg route should be reserved to patients with uncontrolled infection, sepsis, hospitalization, or in those unable to perform SCIg self-administration.

In conclusion, the use of IgRT, long considered only an ancillary therapy in individuals with CLL, must be carefully kept in mind by physicians who manage these patients, given longer life expectancy and increasing use of continuous/long-term therapies (e.g. BCRi and BCL2i) is going to be expected.

## Author contributions

AN and RC contributed to this review’s concept and design, data collection, and wrote the initial draft of the manuscript. MB, VM and GR contributed to study design and review of literature. WB critically reviewed the manuscript. All authors contributed to the article and approved the submitted version.
